# Mental State Examination and Its Procedures—Narrative Review of Brazilian Descriptive Psychopathology

**DOI:** 10.3389/fpsyt.2019.00077

**Published:** 2019-03-05

**Authors:** Helio Gomes Rocha Neto, Carlos Eduardo Estellita-Lins, José Luiz Martins Lessa, Maria Tavares Cavalcanti

**Affiliations:** ^1^Centro Universitario Lusíada, São Paulo, Brazil; ^2^Fundação Oswaldo Cruz (Fiocruz), Rio de Janeiro, Brazil; ^3^Instituto de Psiquiatria, Universidade Federal do Rio de Janeiro, Rio de Janeiro, Brazil

**Keywords:** mental state examination, psychopathology, descriptive psychopathology, history of medicine, psychiatry, diagnosis, diagnosis technics and procedures

## Abstract

**Background:** Mental State Examination (MSE) is compared with physical examination as a reliable method of objective data investigation. There is a growing concern with psychiatric clinics, nosology, and the reliability of diagnostic interview methods as a source of valid diagnostic strategy. Efforts to achieve an international diagnosis protocol have been unsuccessful or polemical. This paper focuses on psychopathology, MSE, and mental function development within Brazilian psychiatry over the last few decades.

**Methods:** Searches, interviews, and narrative reviews were done to look for systematic ways in which to conduct MSE, mental functions, symptom clusters, orientations about data observation and records. Brazilian psychopathology textbooks were examined, if they provided access to consolidated knowledge on psychopathology examination.

**Results:** Sixteen textbooks were selected from a 49 year span. Descriptive psychopathology with phenomenological orientation was the primary trend in the MSE. Concepts derived from different traditions, most lacking common terminology, suggested some divergence among authors. Recommendations for patient observation and how to collect objective data was clear, but MSE standardization efforts were missing. A detailed description of mental function abnormalities was the main MSE record strategy, without consensus about ways to summarize and record this data. In an examination summary, mental strata was divided into “mental functions,” and MSE subsets were frequent. All authors considered the following mental functions: consciousness, perception, thought, memory, attention, orientation, and volition.

**Discussion:** Psychiatric competence demands MSE proficiency. Official documents are not clear about performance and recording standards. MSE data was usually recorded through descriptive psychopathology. A shift from detailed descriptive findings, to an array of observed pathological elements, described through a mental function checklist was observed over time. Clinical practice and research guidelines should consider the development of reliable MSE practices; however, it has been neglected by modern psychiatry/neuroscience through the excessive emphasis on interview protocols. Better MSE practices, and the improvement of bedside skill in psychiatry are necessary and depend on the recovery of psychopathological debates and semiological reasoning, which will allow the return of phenomenology-oriented “observational” techniques.

## Introduction

In modern clinical medicine, a diagnosis is obtained through the crossover of symptoms, obtained by anamnesis or by a clinical interview, and signs of symptoms, obtained through a physical examination and laboratory or image tests (1). The former is supposed to spark multiple hypotheses which the clinician then further investigates by looking at patient's signs and symptoms. Semiology has been described in many textbooks as Porto (2) or Bates (3), and was over time formalized as the paramount method of clinical examination in internal medicine.

Such standard foundational programs were made possible through Claude Bernard's experimental medicine (4), confirmed by Alvan Feinstein one century later, whereby stating that recent clinical epidemiology belongs to the same epistemological strata (5–7). The same method was implemented in most medical specialties, including psychiatry (8, 9). With regard to general medical practice, physical examination (PE), lab, and image procedures provide the standard method to gather objective information, which is then used to refine the previously elaborated hypothesis through the anamnesis/interview method. The very idea of a PE and semiology was strengthened by the transformations of modern medicine, and was consolidated with the Flexnerian reformation of medical schools (10, 11).

Psychiatry has tried to take part in this agenda and match such standards (12). Clinical interviews as a standard procedure for anamnesis in psychiatry, has been rigorously investigated. An extensive bibliography, concerning how to improve an examiners agreement about symptoms, and a rising consensus about the need for a minimal structure for better clinician reliability, is now evident (13–19). Mental health practice often uses Mental State Examination (MSE) as an equivalent to PE from routine clinical examination and as a reliable method for objective data gathering (13, 20–22), since PE and MSE are logically correlated. However, clinical interview/anamnesis is previous to, and also guides PE procedures and laboratory searches, but it is the core of the mental/psychic examination process and used predominately in most cases. However, the interview is a narrative, history taking method, and not an objective sign gatherer tool.

The interview should be a narrative, recollection method, not an objective investigative tool. PE is consistently mentioned throughout almost all propaedeutical textbooks in medicine, with minimal, if not aesthetical, variations. MSE however has not achieved any international protocol or structured general tool, not even a minimal array of standard techniques and clinical report methods. Although MSEs widespread use as a PE correlative might not be suitable and may also be equivocated (9, 23, 24), it still universally used to gather data, objective information and evidence in mental health practice.

MSE was simultaneously developed in different regions of the world, influenced by philosophically-oriented ideas on psychopathology (25, 26). Many psychopathology textbooks have also been written in different languages, according to different traditions, which have resulted in vast variations in technique and nomenclature (27, 28). Nevertheless, mental semiology has been overlooked in most historiographical efforts, despite the importance of nosological history (29).

In the last 30 years, new trends in the history of psychiatry in Brazil developed, but none has considered mental examination. Estellita-Lins attempted to emphasize the phenomenology of living space (espace vécu) in Jaspers Psychopathology Textbook and its vital role concerning signs, “evidence” and examination (30). Cheniaux (31) reviewed some of the Brazilian, and even foreign textbooks about “descriptive psychopathology,” searching for conceptual regularity or terminological “uniformity” among authors, but MSE was not addressed. Viotti Daker worked on the main Brazilian textbook by Nobre de Melo, examining its psychopathological models (32). Again, the MSE was not mentioned, but Melo's emphasis on fully assessing the person, before evaluating the particular functions subdivision, is noteworthy.

There is an increasing concern related to clinics and nosology in psychiatry. This concern might be traced back to Nancy Andreassen's claim concerning the loss of psychopathological knowledge by younger psychiatrists, and to Parnas' Danish group that contested the validity of the schizophrenia nosologic construct in DSM/ICD, further extended to the unreliability of diagnostic interview methods with structured diagnostic questionnaires (33, 34). We should also mention Jacob's questioning of MSE training in India (28), Aragona's interrogation about the collaboration of neuroscience in psychiatric diagnosis, among many others (35). As Rodrigues and Banzato have stated, if a sound agreement concerning the “validity” of a concept in psychiatry had already been achieved, there would not be such confusion around it (36).

We foresee epistemological issues concerning psychiatry and mental health care, that have not yet been resolved, as the importance of examination skills and training in the evidence-based era. These themes are not simply classificatory issues but are fundamental psychopathological efforts demanding a discussion concerning the diagnosis process in mental health and psychiatry.

This study deals with modern psychiatry from a historical perspective but addresses some clinical problems such as MSE, examination reporting, patient records and psychopathology teaching/transmission. A narrow comprehension of what evidence means may have been overlooked such as bedside skills and in particular phenomenological examining tradition (37–39). Maybe ongoing “taxonomic issues” and “classification wars” in psychiatry (40–42) are fair and useful, but we should also pinpoint some relevant matters that concern the examination, clinical reasoning and the diagnostic process itself (43).

Aiming to elucidate the origins, development, and methods of how MSE has been consolidated in Brazil, a review was carried out on the national literature.

## Methods

MSE still lacks proper categorization in MESH and DeCS, and the best results of descriptors or key word searches were related to standard clinical interviews, therefore unsuitable for systematic MSE research purposes. Textbooks are known to be the introductory means to access expert knowledge in the medical field as defined by Ludwig Fleck (44). It was then decided to consider textbooks on psychopathology as a primary reference, following a timeline based on its reference and contents. A backward reference search was carried out at the start of 2017, using the Universidade Federal do Rio de Janeiro Library System, to look for psychopathology textbooks written or edited by Brazilian authors (UFRJ, Rede Minerva).^1^ The same search strategy was extended to main bookstore digital systems, and sites specialized in old or out of print editions, and finally to Google and Google Scholar. The main search string used was “psicopatologia” or “psiquiatria.”

## Results

An initial list of 35 textbooks was selected. Among them were textbooks translated into Portuguese, not edited or written by Brazilian authors, and which were therefore excluded. Other books without any relevant chapters on MSE were also excluded. Twelve textbooks were finally chosen for the present study. The original list of books found and selected are presented in Tables 1, 2. Whenever possible, the first and last edition of each book was consulted, and discussions about systematic ways to conduct MSE, the ordering of MSE topics (distinct functions), orientations about data gathering and registration, definitions about “mental functions” and its levels, symptom clusters, groupings and set organization, were studied. Referenced articles and books citations were also checked. In older textbooks, terms and notions that are semantically related to MSE have changed over time. Therefore, any mention of a mental examination that clearly described careful mental observation techniques or descriptions was considered as similar or equivalent to MSE.

**Table 1:** Textbook set with editions, publication years, and the 12 titles (in bold) chosen for investigation of MSE.

**Textbook**	**Author/editor**	**Edition**	**Publication year**
**1. Psychopathology course**^a^ **(Curso de psicopatologia)**	Isaías Paim	1st, 11th	1969, 1993
**2. Treaty of psychiatry**^a^ **(Tratado de psiquiatria)**	Augusto Luiz Nobre de Melo	1st, 3rd	1970, 1981
**3. Treaty of clinical psychiatry**^a^ **(Tratado de clínica psiquiátrica)**	Isaías Paim	1st	1976
**4. Diagnoses in psychiatry**^a^ **(Diagnóstico em psiquiatria)**	José Leme Lopes	1st	1980
**5. Psychopathology fundamentals**^a^ **(Fundamentos de psicopatologia)**	Luiz Salvador de Miranda Sá Jr	1st	1988
**6. Psychopathology and semiotics of mental disorders**^a^ **(Psicopatologia e semiologia dos transtornos mentais)**	Paulo Dalgalarrondo	1st, 2nd	2000, 2008
**7. Basic psychiatry**^a^ **(Psiquiatria básica)**	Mario Rodrigues Louzã Neto and Helio Élkis	2nd	2007
**8. Manual of psychopathology**^a^ **(Manual de psicopatologia)**	Ellie Cheniaux	4th, 5th	2011, 2015
**9. Manual of psychic exam: an introduction to psychopathology**^a^ **(Manual do Exame Psíquico uma Introdução a Psicopatologia)**	Claudio Lyra Bastos	3rd	2011
**10. Compendium psychiatric clinic**^a^ **(Compêndio de Clínica psiquiátrica)**	Forlenza and Miguel	1st	2012
**11. Basic psychopathology and psychiatry**^a^ **(Psicopatologia e psiquiatria básicas)**	Geraldo José Ballone, José Carlos Souza, and Liliana Andolphi M. Guimarães	2nd	2013
**12. Manual of descriptive psychopathology and psychiatry semiology**^a^ **(Manual de psicopatologia descritiva e semiologia psiquiátrica)**	Leonardo F. Fontenelle and Mauro V. Mendlowicz	1st	2017
13. Psychopathology contributions and psychiatry clinic^a^ (Psicopatologia Contribuições a Clínica Psiquiátrica)	Cleto Brasileiro Pontes	1st	2000
14. Abnormal psychology: clinical perspectives on psychological disorders (Psicopatologia)	Susan K. Whitbourne	7th	2015
15. Psychopathology: theory and clinic^a^ (Psychologie pathologique: théorique et clinique)	Jean Bergeret	19th	2004
16. Scheduled meeting with madness: teaching and learning psychopathology^a^ (Encontro marcado com a loucura: ensinando e aprendendo psicopatologia)	Tania Cocciufo	1st	2008
17. Psychopathology: fundamentals, disorders, and consequences of chemical addiction^a^ (Psicopatologias: fundamentos, transtornos e consequencias da dependencia química)	Rita Campos Ferreira	1st	2015
18. Teaching-learning of psychopathology: a colective project^a^ (Ensino-aprendizagem de psicopatologia: um projeto coletivo)	Ligia Maria Ananias Cardoso	1st	2004
19. Psychopathology of daily clinic^a^ (Psicopatologia da clínica cotidiana)	Gustavo Fernando Julião de Souza	1st	2015
20. Foundations of psychopathology	John C. Nemiah, Kenneth Appel	1st	1981
21. Manual of general psychopathology^a^ (Manual de psicopatologia general)	Pedro J. Mesa, Pedro J. Mesa Cid, and Juan F. Rodriguez Testal	1st	2010
22. Manual of psychopathology^a^ (Manual de psicopatologia)	Diogo Telles correia	1st	2013
23. Phenomeno-structural psychopathology^a^ (Psicopatologia fenomeno estrutural)	Anna Elisa de Villemor Amaral	1st	2010
24. Psychiatry for general doctor^a^ (Psiquiatria para o medico generalista)	Gustavo C. Mansur	1st	2013
25. Psychoanalytic clinics of contemporaneous psychopathology^a^ (A clinica psicanalitica das psicopatologias contemporâneas)	Gley P. Costa	1st	2007
26. Adolescence et psychopathologie^a^ (Adolescência e psicopatologia)	Daniel Marcelli and Alain Braconnier	8th	2013
27. Homeopathic practices in psychopathology (Pratique homéopathique en psycho-pathologie)^a^	Jacqueline Barbancey	1st	1981
28. Introduction to psychopatology^a^ (Introduction à la psychopathologie)	Jean Ménéchal	1st	2007
29. Lived time: phenomenological and psychopathological studies (Le Temps vécu. Étude Phénoménologique et Psychopathologiques)	Eugéne Minkowski	1st	2013
30. Abnormal psychology: an integrative approach	David H. Barlowe and V. Mark Duran	8th	2018
31. Evolutionary psychopathology^a^ (Psicopatologia evolutiva)	Francisco B. Assumpção Jr.	1st	2008
32. Fundamental psychopathology research^a^ (Pesquisa em psicopatologia fundamental)	Edilene Freire de Queiroz, Antonio Ricardo Rodrigues da Silva	1st	2002
33. Conceptual psychopathology^a^ (Psicopatologia conceitual)	Adriano C.T. Rodrigues, Luis Guilherme Streb, Maurício Viotti Daker, Octavio Domont de Serpa Júnior	1st	2012
34. The black book of contemporary psychopatology^a^ (El libro negro de la psicopatologìa contemporánea)	Alfredo Jerusalinsky, Silvia Frendrik	1st	2011
35. Phenomenological contemporary psychopathology^a^ (Psicopatologia fenomenológica contemporanea)	Guilherme Peres Messas	1st	2008

a *Author translation*.

**Table 2:** MSE in the investigated textbooks, number of items, how it is named, and their items or assessed functions.

**Textbook**	**N° of items**	**How MSE is named**	**Items**
Psychopathology Course^a^ (Curso de Psicopatologia) Isaías Paim, 1st edition, 1969	12	No term	1. Perception Disturbances—Illusions and basic perception phenomena (Alterações da Percepção) 2. Representations Disturbances—Hallucinations and Imagination (Alterações das Representações) 3. Concepts—Think Process (Alterações dos Conceitos) 4. Judgments—Delusions, but also insight and Reasoning (Alterações do Juízo) 5. Reason—Flow of Thinking, but also Thinking Process and Associative Process (Alterações do Raciocínio) 6. Disturbances of Memory (Alterações da Memória) 7. Disturbances of Attention (Alterações da Atenção) 8. Disturbances of Orientation (Alterações da Orientação) 9. Disturbances of Consciousness (Alterações da Consciência) 10. Disturbances of Affect and Emotions (Alterações da Afetividade) 11. Disturbances of Volition and Execution (Alterações da Atividade Voluntária) 12. Disturbances of Speech and Language (Alterações da Linguagem)
Treatise of Psychiatry^a^ (Tratado de Psiquiatria) Augusto Nobre de Melo, 1st edition, 1970	6	No Term	1 Consciousness and Its Abnormalities: Attention, Orientation—Self Experience, Time Experience, Temporal Experience (Da Consciência e suas Perturbações: Atenção, Orientação—Consciência do Eu, Vivências Temporais e Espaciais) 2 Intelligence and Its Abnormalities (Da Inteligência e suas Anormalidades) 3 Elementary Intellectual Activity: Perception and Memory (Atividades Intelectuais Elementares: Percepção e Memória) 4 Superior Intellectual Activity: Comprehension, Ideation, Imagination, Association, Thinking, Judgment, Reasoning, Expression (Atividades Intelectuais Superiores: Compreensão, Ideação, Imaginação, Associações, Pensamento, Juízo, Raciocínio e Expressão) 5 Psychopathology Affect: Emotions and Feelings (Psicopatologia da Afetividade: Emoções e Sentimentos) 5.1 Primary Emotions (Emoções Primárias): Shock (Choque), Choleric Affect (Emoção Colérica), Affectionate Affect (Emoção Afetuosa); 5.2 Secondary Emotions (Emoções Secundárias): Sensorial Affective State (Estados Afetivos Sensoriais),Vital Affective State (Estados Afetivos Vitais); Derivative Emotions (Emoções Derivadas e Mistas), Soul and Spiritual Feelings (Sentimentos Anímicos e Espirituais); Passion and Tendencies (Inclinações e Paixões) 6 Volition and Execution Psychopathology (Psicopatologia da Vontade): 6.1 Elementary Automatism: Acquired Automatism, Voluntary and Involuntary Activity (Automatismos Elementares: Adquiridos, Voluntárias e Involuntárias) 6.2 Volition Nature (Natureza da Vontade) 6.3 Volition Anomalies (Perturbações da Vontade): Pathological Impulse (Impulsos Patológicos), Perversions and Compulsions (Compulsões e Perversões), Alterations of Psychomotricity (Alterações da Psicomotricidade)
Treatise of Clinical Psychiatry^a^ (Tratado de Clínica Psiquiátrica) Paim, 1st edition, 1976	14	Psychic exam	Does not establish a clear division between mental functions, but describe 14 segments to analyze
Diagnoses in Psychiatry^a^ (Diagnóstico em Psiquiatria) Leme Lopes, 1st edition, 1980	9	Psychic Exam	1. Presentation (Apresentação) 2. Consciousness – Dimensions; Orientation: Time, Space and Person; Attention (Consciência—Nível; Orientação: Autopsíquica, Alopsíquica e somatopsíquica; Atenção) 3. Disturbance of Memory (Memória) 4. Disorders of Perception (Percepção) 5. Affect and Emotional Disorders (Afetividade) 6. Thinking—Process and Beliefs (Pensar—Forma e Conteúdo) 7. Psychomotricity—Motricity, Volition (Psicomotricidade—Motricidade, Volição) 8. Personality (Personalidade) 9. Insight
Psychopathology Fundamentals^a^ (Fundamentos de Psicopatologia) Miranda Sá Jr, 1st edition, 1988	9	Mental Exam	1. Ambient description and Personal Presentation (Descrição do Paciente e Condições Ambientais) 2. Appearance and Behavior (Aspecto Geral e Comportamento Espontâneo) 3. Attitude and Cooperation (Atitude Frente ao Exame) 4. Cognition: Perception, Attention, Thinking, Imagination (Estado da Cognição: Sensopercepção, Atenção, Pensamento, Imaginação) 5. Affect: Volition,Affect and Emotions (Afetividade: Pulsões, Emoções e Sentimentos) 6. Psychomotricity: Motor Activity and Language (Estado da Motricidade: Psicomotricidade, Expressão e Linguagem) 7. Consciousness—Vigilance (Estado da Consciência—Vigilância) 8. Memory (Estado da Memória) 9. Orientation (Estado da Orientação)
Psychopathology Course^a^ (Curso de Psicopatologia) Isaías Paim, 11th edition, 1993	13	No term	1. Perception—Illusions and basic perception phenomena (Alterações da Percepção) 2. Representations—Hallucinations and Imagination (Alterações das Representações) 3. Concepts—Thinking Process (Alterações dos Conceitos) 4. Judgments—Beliefs, but also insight and Reasoning (Alterações do Juízo) 5. Reason—Think Flow, but also Thinking and Associative Process (Alterações do Raciocínio) 6. Disturbances of Memory (Alterações da Memória) 7. Disturbances of Attention (Alterações da Atenção) 8. Disturbances of Orientation (Alterações da Orientação) 9. Dimensins of Consciousness (Alterações da Consciência) 10. Affect and Emotions (Alterações da Afetividade) 11. Volition and Execution (Alterações da Atividade Voluntária) 12. Life Tendencies—Volitional Instincts (Alterações das Tendências Vitais) 13. Language and Speech (Alterações da Linguagem)
Psychopathology and Semiotics of Mental Disorders^a^ (Psicopatologia e Semiologia dos Transtornos Mentais) Paulo Dalagalarrondo 1st edition, 2000; 2nd edition 2008	14	Psychic exam/MSE	1. Consciousness and its Disturbances (A Consciência e suas Alterações) 2. Attention and its Disturbances (A Atenção e suas Alterações) 3. Orientation and its Disturbances (A orientação e suas Alterações) 4. Disturbances of Memory (Memória) 5. Intelligence (Inteligência) 6. Language and Speech (Linguagem) 7. Affect and Emotions (Afetividade) 8. Volition (Vontade) 9. Execution (Psicomotricidade) 10. Personality (Personalidade) 11. Perception (Sensopercepção) 12. Thinking—Flow, Process and Beliefs (Pensamento) 13. Insight (Juízo de Realidade) 14. Self-awareness (Vivência do Eu)
Manual of Psychopathology^a^ (Manual de Psicopatologia) Elie Chenieux, 1st edition, 2002; 3rd edition, 2005; 4th edition, 2008;4th edition, 2011; 5th edition, 2015	18	Psychic exam/MSE “Súmula psicopatológica”	1. Appearance (Aparência) 2. Attitude (Atitude) 3. Consciousness—Vigilance and Dimensions (Consciência—Vigilância) 4. Attention (Atenção) 5. Perception (Sensopercepção) 6. Memory (Memória) 7. Language (Linguagem) 8. Thinking—Flow, Process and Beliefs (Pensamento) 9. Intelligence (Inteligência) 10. Imagination (Imaginação) 11. Volition (Conação) 12. Pragmatism (Pragmatismo) 13. Psychomotricity (Psicomotricidade) 14. Affectivity—Emotions and Affect (Afetividade) 15. Time, Space and Personal Orientation (Orientação Alopsíquica) 16. Self-awareness (Consciência do Eu) 17. Future Projects (Prospecção) 18. Insight (Consciência de Morbidade)
Basic Psychiatry^a^ (Psiquiatria Básica) Louzã Neto and Helio Élkis, 2nd edition, 2007	15	Psychic exam/ MSE “Súmula psicopatológica”	1. Appearance (Aspecto Geral) 2. Dimensions of Consciousness (Nível de Consciência) 3. Time, Space and Personal Orientation (Orientação) 4. Attention (Atenção) 5. Memory (Memória) 6. Perception (Percepção Sensorial) 7. Thinking—Flow, Process, and Beliefs (Pensamento) 8. Language and Speech (Linguagem) 9. Reality Reasoning (Juízo da Realidade) 10. Affect and Emotions (Vida Afetiva) 11. Volition (Volição) 12. Psychomotricity (Psicomotricidade) 13. Intelligence (Inteligência) 14. Personality (Personalidade) 15. Transference (Sentimentos Contra Transferenciais) 16. Insight (Crítica em Relação aos Sintomas e Desejo de Ajuda)
Manual of Psychic Exam: An Introduction to Psychopathology^a^ (Manual do Exame Psíquico uma Introdução a Psicopatologia) Lyra Bastos, 3rd edition, 2011	13	Psychic exam “Súmula psicopatológica”	1. Dimensions of Consciousness (Estado de Consciência) 2. Attention (Atenção—Vigilância e Tenacidade) 3. Appearance/Presentation (Aspecto Geral) 4. Attitude and Cooperation (Atitude em Relação ao Entrevistador) 5. Psychomotricity and Behavior (Comportamento e Psicomotricidade) 6. Language and Speech (Linguagem) 7. Time, Space and Personal Orientation (Orientação) 8. Affect and Emotions (Afetividade) 9. Volition and Pragmatism (Vontade e Pragmatismo) 10. Thinking—Flow, Process, and Beliefs (Pensamento) 11. Perception (Sensopercepção) 12. Memory (Memória) 13. Intelligence (Inteligência)
Compendium of Psychiatric clinic^a^ (Compêndio de Clínica Psiquiátrica) Forlenza and Miguel, 1st edition, 2012	9	MSE	1. Consciousness and Attention (Consciência e Atenção) 1.1 Self-awareness (Consciência do eu) 1.2 Dimension of Consciousness (Vigília) 1.3 Time, Space and Personal Orientation (Orientação) 1.4 Cognition (Cognição) 1.5 Awareness (Apercepção) 1.6 Responsiveness (Responsividade) 1.7 Alertness (Alerta) 1.8 Attention (Atenção) 1.9 Mental Activity Course (Curso da Atividade Mental) 2. Memory (Memória) 3. Inteligence (Inteligência) 4. Perception (Alterações da Sensopercepção) 4.1 Imagination (Imaginação) 4.2 Representation (Alterações da Representação) 4.3 Temporal and Spatial Experience (Tempo e Espaço: Vivência e Rendimento) 5. Thought and Language (Pensamento e Linguagem) 6. Reasoning (Juízo) 7. Affect (Afetividade) 8. Volition: Impulse, Instinct and Will (Volição: Impulso, Instinto e Vontade) 9. Movement (Movimento)
Basic Psychopathology and Psychiatry^a^ (Psicopatologia e Psiquiatria Básicas) Geraldo José Ballone, José Carlos Souza, and Liliana Andolphi M. Guimarães, 2nd edition, 2013	11	MSE	1. Appearance and General Behavior (Aparência e Comportamento Durante o Exame) 2. Relationship with Interviewer (Relação com o Entrevistador) 3. Attention and Consciousness (Consciência e Atenção) 4. Time, Space and Personal Orientation (Orientação) 5. Thinking—Flow and Beliefs (Pensamento) 6. Memory (Memória) 7. Affect and Emotions (Afetividade) 8. Perception (Sensopercepção) 9. Volition (Vontade) 10. Psychomotricity (Psicomotricidade) 11. Intelligence (Inteligência)
Manual of Descriptive Psychopathology and Psychiatry Semiology^a^ (Manual de Psicopatologia Descritiva e Semiologia Psiquiátrica) Leonardo F. Fontenelle and Mauro V. Mendlowicz, 1st edition, 2017	16	MSE “Súmula psicopatológica”	1. Appearance (Aparência) 2. Nonverbal Communication (Comunicação Não Verbal) 3. Verbal Communication (Comunicação Verbal) 4. Consciousness (Consciência) 5. Attention (Atenção) 6. Time, Space and Personal Orientation (Orientação) 7. Self-consciousness (Consciência do Eu) 8. Intelligence (Inteligência) 9. Memory (Memória) 10. Thinking—Reasoning, Process and Flow (Pensamento) 11. Language and Speech (Linguagem) 12. Perception (Sensopercepção) 13. Imagination (Imaginação) 14. Needs/Volition (Necessidades) 15. Affect and Emotions (Afetividade) 16. Psychomotricity (Psicomotricidade)

a *Authors translation*.

*Mental functions translated by the author*.

A comprehensive review was carried out on Brazilian authors, researchers and literature, focusing on different MSE traditions, describing its various approaches and MSE segmentation around psychic functions. The list of discrete elements assembled into MSE was studied in detail. A structured method to register significant psychopathological abnormalities/anomalies through examinations did show up in the search results. It was identified as “súmula psicopatológica” (psychopathological summary) in some textbooks.

The works examined spanned over the last 49 years. The books were written mostly by psychiatrists with clinical expertise, working as University Professors or lecturers. Most publications were designed to teach psychopathology to medical doctors being trained as a resident/internist in psychiatry. No MSE standard was identified, even though every author confirmed its relevance and consistency.

Most authors provided orientations on how to proceed with a careful patient observation during an interview, aimed at obtaining objective data. The most referred method, to organize and register MSE, consistently cited by some authors, was descriptive psychopathology with a phenomenological orientation. Textbooks agreed and actively encouraged a very detailed description of observed mental functions as the best way to record MSE. Additionally, there was no agreement on how to summarize its components (20–22, 45–54).

Throughout the time span studied, MSE was not the standard term used to refer to psychic examination and was first adopted in 2000 in the Brazilian psychopathology and semiology textbook by Paulo Dalgalarrondo (46), after which it was consistently used. It remains unclear if the term was chosen because of evidence-based efforts on examination, or if it represents a particular tradition. Regardless, Luiz Salvador de Miranda Sá Jr mentioned “mental examination,” an expression semantically related (50), and Elias Paim called it “psychic examination” around 1976, as did Leme Lopes in about 1980 (51, 55). The only author that did not use MSE after 2000 was Claudio Lyra Bastos, who still mentioned “Psychic Examination” (47).

All authors used subdivisions to analyze and describe MSE, but each provided a specific set. Even though there was a clear consensus that consciousness could only be artificially fragmented for didactic purposes, no agreement, convergence or discussion about the nature or number of necessary items could be identified. The theoretical basis underlying the operating subdivisions is therefore lacking. It remains unclear how these mental strata turned into a divided set of “mental functions.” Table 2 shows the items that form MSE, according to each author.

A concise examination summary through “súmula psicopatológica” was mentioned by only four authors (21, 22, 45, 47, 48). It was not possible to track the origins of this notion, since the authors did not mentioned or contextualize it. Some hypothesis is developed through discussion.

It is noteworthy that among the initial textbook list, some belonged to a field aimed at researching psychotherapy and psychoanalysis—therefore promoting a broader signification of psychopathology. Although these books were not included, and considering that psychoanalytic psychopathology is different from descriptive psychopathology, it probably suggests that psychoanalysis has had some influence in Brazilian psychiatric practices, perhaps in a slightly different way than what occurred in the United States (33, 56, 57). The term “interview” has widespread use when referring to the diagnostic and therapeutic encounter with patients, including psychoanalytical sessions, which may add further confusion when searching for Psychiatric methods of MSE using indexed expressions, and which justifies the frequency of these textbooks in the initial search sample. A better evaluation of such influence would be desirable, but it is out of the scope of this paper.

## Discussion

Mental Health as a Brazilian tradition started in the nineteenth-century, after the creation of the hospice, by a Portuguese emperor (58–61). It was not before the beginning of 1900, however, that academic psychiatry began to thrive, particularly after studies published by Juliano Moreira, Ulisses Pernambucano, followed by studies on forensic psychiatry published by Nina Rodrigues and Franco da Rocha psychiatry (62–64).

The first psychopathology textbook edited by a Brazilian author was published by Isaías Paim at the very end of the 1960s (52), as far as we were able to trace. Paim's textbook stresses that psychopathology is not a psychiatric “tool,” but an entirely different “science,” that could be applied by psychiatrists to comprehend and investigate mental illness (52). Influenced by German and French authors, Paim thoroughly recommended detailed observations and descriptions of subjective reports (52) and behaviors as the best way to register and carry out psychic examination (55). This coupling of observation and description was continually reinforced by most authors analyzed (20–22, 45–55, 65).

The vast majority of textbooks considered MSE with clear subdivisions, except Paim's (55) Psychiatry Treatise. The number of partitions ranged from 6 (49) up to 18 items (45). The first time they are referred to as “Mental Functions” in our textbook sample was in Dalagalarrondo's “Psicopatologia e Semiologia” (46). Despite considerable variations in mental function descriptions and items adopted, all authors have explicitly considered the following mental functions: consciousness, perception, thought (not frequently comprising its 3-fold structure—Flow, Process, and content/Beliefs), memory, attention, orientation, and volition. Paim did not list mental functions in his treatise but had openly used it in his 1969 psychopathology textbook. These conceptual developments in Brazilian psychopathology call for further studies. We considered them as hints at the importance of mental function fragmentation and structure in psychopathology.

It was not possible to identify how subdivisions were adopted or created according to Brazilian textbooks. The lack of theoretical background may be responsible for the significant variation observed. Dividing or stratifying MSE seems to be the best method to analyze and to conveniently engage in description efforts, to evaluate psychic life. A discussion concerning ways to split MSE for adequate observation and educational purposes is out of the scope of the present review, but highly desirable. Since there are no globally standardized guidelines on psychopathology training (66), bedside examination skills must be regarded seriously, as an asset worth further exploring.

This chronologically considered textbook series, suggests that the first Brazilian authors that wrote about psychopathology—Nobre de Melo, (49) and Isaías Paim, (52)—did not aim to or were not used to describe MSE as mental function subdivisions, but were eager to build long and meticulous reports of observed behavior, through a personal interaction with patients. Contemporary Brazilian authors however usually mention clear descriptions of mental function subdivisions, setting standards on how to study MSE and how to describe it. It was not possible to identify a “standard” MSE organization, although a written description of all that is observed is consistent in both older and recent textbooks (20–22, 45–48, 50, 51, 53–55, 65). We could not identify any efforts aimed toward comparative clinical psychopathology.

The origins of the clinical resume called “súmula psicopatológica” could not be identified. It may have a forensic and juridical background, as its etymology suggests. Besides lawsuit writing rights, there were many other compulsory examination practices that the Brazilian and then the eugenics Constitution (1937) recommended. Psychiatrists were accountable for defining a person's state of mental health in many common judiciary cases such as criminal subjects, couples before marriage, institutionalized children, among others. For instance, forensic demands synthesis during a long diagnostic process and extensive judicial records.

Some leading psychiatrists, such as Franco da Rocha were very fond of this reporting procedure after clinical examination. A historiographic article, reviewing old patient files, depicts an explicit use of “súmula” by psychiatrists (67–69) when writing down their patient's observations around 1929. It is then possible to affirm that the use of “súmula” as the name given to the set of observed signs and symptoms in psychiatric interview was already widespread in Brazilian psychiatry by the early twenties.

Within the textbook set investigated, the first register of the word “súmula”—as a concise list of mental function subdivisions, containing psychopathological disturbances, observed through MSE—appears in Dalgalarrondo's textbook (46). The term “súmula psicopatológica” was also mentioned in Cheniaux psychopathology treatise (70). Cheniaux's communication, however, admitted that this precise notion had been in colloquial use since the early 90s, and declared that he was first introduced to it by prof Dr. Miguel Chalub, who also introduced him to categorized lists of mental functions with descriptive purposes^2^.

Chalub, in turn, declared that he learned this exact expression from Professor José Leme Lopes, the chairman of UFRJ Psychiatric Institute in the seventies.^2^ However, Leme Lopes' “Diagnoses in Psychiatry,” published in 1980 does not contain any mention of it (51). Neither Chalub nor Cheniaux needed to trace its roots but acknowledged its common use in bedside practices. Both authors hypothesized that it was borrowed from international psychopathological tradition, and that it become mainstream through everyday use in many institutional facilities in Brazil. The same inference about the expression “mental function” might therefore be adopted.

Efforts to standardize medical examinations have been at the core of scientific experimental medicine. These attempts have branched alongside psychopathology developments. The systematization of diagnostic psychiatric interview has been considered a significant step toward the improvement of clinician reliability. However, doubts about the best way to use it clinically, still remains (15, 71, 72). Countries, like Denmark, have already regulated the use of at least one standard interview in clinical practice, for any diagnosis in psychiatry (73) and others such as Australia use diagnostic tools in a mental health triage (74, 75). These are anamnesis/interview standardized methods however, not MSE.

ICD and DSM contain examining tools (Schedules for Clinical Assessment in Neuropsychiatry—SCAN, Structured Clinical Interview for DSM-5 Disorders—SCID-5, Mini International Neuropsychiatric Interview—MINI) (76–78). OPCRIT is supposedly useful to organize MSE, although it seems vastly different from what is usually accepted in descriptive psychopathology, since it provides neither mental function subdivisions nor any coordinated step care to enhance MSE observation and description (79). Nevertheless, the entire MSE procedure demands more than a structured interview, since it is not analogous to anamnesis, but correlated to the PE procedure. We suppose that MSE is not entirely congruent or wholly embedded in semi-structured interview protocols. Attempting to turn personal experiences into objective data is probably a source of unreliability between examiners, as previously described (19, 80–84). As far as we know, there is no standard method or procedure for MSE.

Parnas argues that DSM-V and ICD-10 were constructed to avoid subjectivity, but its developers have not accomplished such intent (23). He emphasizes that it is easy to find objective, observable signs inside many diagnostic criteria, such as “blunt affect” in schizophrenia or “fast speech” in mania. Such categories should have been avoided in a categorial diagnostic system based on a standard interview, that was developed to eliminate examiner opinion as a source of unreliability. In a standard interview, all diagnostic emphasis relies on a “yes” and “no” type series of questions, directed at the patient, who decides if a symptom is or is not present. If MSE categories were to be used in a categorial diagnostic system, it should provide a template MSE method to be followed by the examiner during practice. A list of valid abnormal psychopathological categories for classificatory issues should also be provided.

In Brazil, at least one recent official report, demands a careful description of MSE subdivisions into patient record files (85). The official document on psychiatric training in Brazil states that all candidates must be proficient on MSE skills (apply and record). Despite that, a standard MSE is not explicitly provided, and it is not clear how it should be done or registered (86). The Brazilian Association of Psychiatry (ABP), the professional body responsible for education, training, setting and raising standards in Brazilian Psychiatry, did not mention or provide any statements concerning MSE, until now (4th of May 2018).^3^

Since 2014, doctors that have experience in psychiatry or that have attended a psychiatry internship and desire to obtain a certificate in psychiatry must be submitted to a practical clinical examination test, in which they are observed while interviewing and examining a patient.^3^ It is not evident in the documentation how the MSE, or even the clinical interview, would be assessed and what they are expected to perform during a practical examination test (87). The only clue provided is from Dalgalarrondo's textbook inclusion of bibliographic references, which suggests that a detailed descriptive MSE and “súmula psicopatológica” is expected from candidates.

## Conclusion

Our research suggests that descriptive psychopathology seems to be the usual MSE method used to observe and record data, not much different from the European schools or other parts of the world (80). Seven mental functions were consistently identified in the selected textbooks (consciousness, perception, thought, memory, attention, orientation, and volition), however no standard MSE and mental functions set was found. It was possible to identify a shift from the semiological discussion in Brazil during the last 50 years, from a detailed descriptive routine observed during the patient interaction to an array of observed pathological elements described through a mental function checklist. The “súmula psicopatológica” appears to be one pattern of examination, which could be improved or updated. Ethnopsychiatry or transcultural psychiatry research is needed regarding MSE, to achieve a regional attunement with patients and to comprehend the MSE practice in general.

MSE altogether, with anamnesis or a clinical interview, provides the basis for psychiatric clinics and research. Good clinical exercise and research guidelines in psychiatry must include the development of reliable MSE practice; however, it seems to have been neglected by modern psychiatry and neuroscience. Stressing interview protocols might flatten examination skills and impoverish MSE abilities. Development of better MSE practices and the improvement of bedside skills in psychiatry rely on reviving the psychopathological debate and semiologic reasoning of a vibrant tradition, and allowing for the return to a phenomenology-oriented “observational” technique.

Since we are now dealing with knowledge that has almost become lost, the recovery of the history of psychiatry and of national/regional practices plays a paramount role in bringing previous experiences to the foreground, that could assist in the pursuit of this everlasting objective. In other words, the history of psychiatry plays a critical and hermeneutical role, and particular national attempts and enterprises in psychopathology should be re-evaluated. Practice standardization is now an international goal, but should not lead to one-way, top-down unification from high-tech oriented research centers, as global mental health policies have already advised. A call for diverse, multiple and manifold cultural experiences in MSE is necessary for the future development and improvement in Psychiatric practice and research.

## Limitations

This is a comprehensive review about Psychopathology Textbooks in Brazil and, although rigorous work has been done, it is possible that some critical publication is lacking due to methodological bias. Furthermore, because we dealt with vintage books, which have not yet been cataloged by electronic repositories, it is possible that old Brazilian Textbooks with some contradictory information were not found. Mainstream psychiatry has neglected psychiatric semiology, Mental State Examination, Mental Functions and its role in psychopathology standards and clinical practice, so it is possible that other authors have already answered the questions presented here, which was not picked up by our search strategy. This is a very complicated issue that needs to be addressed by a multi-professional team of linguists, anthropologist, science and medicine historians, and psychiatry practitioners. Further work may clarify and better elucidate these questions and issues.

## Author Contributions

HR elaborated the search strategy, compiled the main data, and written the text. CE-L reviewed the compiled data, added most of historical data, and reviewed the main text. JL contributed for the elaboration of search strategy, delineated the main objective, and reviewed the final version of the text. MC contributed as main reviewer of data gathering, text writing, and conclusion.

### Conflict of Interest Statement

The authors declare that the research was conducted in the absence of any commercial or financial relationships that could be construed as a potential conflict of interest.

## Footnotes

^1^https://minerva.ufrj.br

^2^Personal Communication.

^3^http://www.abp.org.br/portal/
